# Quantifying canine interactions with smart toys assesses suitability for service dog work

**DOI:** 10.3389/fvets.2022.886941

**Published:** 2022-09-02

**Authors:** Ceara Byrne, Thad Starner, Melody Jackson

**Affiliations:** ^1^Traverso Lab, Massachusetts Institute of Technology, Boston, MA, United States; ^2^Animal Centered Computing Lab, Georgia Institution of Technology, Atlanta, GA, United States

**Keywords:** quantified interactions, computational behavior, Canine Companions for Independence, animal behavior, instrumented toys

## Abstract

There are approximately a half million active service dogs in the United States, providing life-changing assistance and independence to people with a wide range of disabilities. The tremendous value of service dogs creates significant demand, which service dog providers struggle to meet. Breeding, raising, and training service dogs is an expensive, time-consuming endeavor which is exacerbated by expending resources on dogs who ultimately will prove to be unsuitable for service dog work because of temperament issues. Quantifying behavior and temperament through sensor-instrumented dog toys can provide a way to predict which dogs will be suitable for service dog work, allowing resources to be focused on the dogs likely to succeed. In a 2-year study, we tested dogs in advanced training at Canine Companions for Independence with instrumented toys, and we discovered that a measure of average bite duration is significantly correlated with a dog's placement success as a service dog [*Adjusted OR* = *0.12, Pr(*>*|z|)* = *0.00666*]. Applying instrumented toy interactions to current behavioral assessments could yield more accurate measures for predicting successful placement of service dogs while reducing the workload of the trainers.

## Introduction

A *service dog* is a dog that is specifically trained to aid a person with a disability (1, 2). There are upwards of 500,000 active service dogs in the US at present time. To become a service dog, candidates go through ~2 years of extensive training. Depending on their program and the career they are best suited for, raising and training costs can reach up to $50,000 per candidate (3). Programs like Canine Companions for Independence (CCI), who breed their dogs specifically for temperament suitable for service dogs, still incur significant cost. Even with CCI's large breeding and puppy raiser program, as many as 60% will fail in training due to behavioral issues. Identifying quantifiable features and “profiles” for which dogs are likely to succeed or fail in their program as early as possible has the potential to increase availability and save millions of dollars in training and living expenses. This goal is important specifically because CCI is a nonprofit, and these dogs are either gifted to their recipients or are sold at a loss to the centers that train them.

Traditional predictors of training success for service dogs (detailed in section Existing research on behavioral testing) require subjective methods of temperament assessment. *Temperament* is the inherent nature of a dog, which affects (often unalterably) their behavior (4). For example, a dog's temperament can be generally calm, or fearful, or aggressive.

Currently, the accuracy of generalizable behavioral evaluations have been shown to range between 64 and 87% (5). And while specificity, which is the true negative rate and highlights correctly identifying dogs who should fail out, is somewhat consistent, ranging between 81.8 and 99.6%, the sensitivity, which is the true positive rate and looks at correctly identifying which dogs should be placed, varies between 3 and 85% (5). The problem we address in this research is to strengthen the consistency of the true positive rate of identifying dogs who should successfully be placed as service dogs and, hopefully, in identifying for which programs they would be best suited for.

Due to this variability in accuracy and true positive rate, veterinarians and animal behaviorists are calling for robust quantitative measures of canine behavior and interaction (6–9). In search of a quantifiable measure of temperament, researchers have identified four components to measure the validity of a temperament test: the test must be (1) conducted and (2) evaluated consistently across all participants; (3) it must be reliable and, ideally, replicable with significant correlations; and (4) it must accurately measure what the experimenter is attempting to measure—in other words, it must exhibit internal validity (10, 11).

In Byrne (12), we discussed the construction of instrumented toys for predicting suitability of service dogs. Although the prediction was effective (87.5% accurate) and would save CCI over five million dollars a year in resource costs, we had not yet delved into understanding the factors that made the predictions so accurate. In this new study, we investigate if the quantified toy interactions have any explanatory effects on the outcomes of the service dogs. As John Spicer says, “it is possible to make successful predictions without being able to explain why these predictions work. Similarly, the workings of a phenomenon may well be explained, but predicting its future states may be impossible because of the many other factors that enable or prevent the occurrence of these states” (13). In this article, our research investigates *why* our predictions work and provides an understanding of which computational play-based interactions are indicative of service dog suitability.

## Existing research on behavioral testing

The rate of success for most service-dog-in-training programs hovers around 30–50% of dogs entering a program (14–16). To improve these numbers, assorted subjective behavioral tests have been leveraged over the past 80 years by service dog groups and breeders to varying degrees of success (17–21). Recently, researchers have even employed biometric means such as imaging dogs' brains with fMRI (14, 22) and eye-tracking (22) to obtain behavioral information. Due to behavioral variability in canines, the animal behaviorist community has not been able to standardize a specific vocabulary fully describing the complexity of behaviors. Even the label “temperament“ has been defined differently across researchers. For example, in their survey of the literature, Diederich and Giffroy (11) compared the relationships across the definitions of temperament and stated that “it implies that these differences (in temperament) are: (1) present at an early age; (2) elicited in a set of situations; (3) (relatively) stable over time.”

To assess temperament, evaluators observe canine responses to objects and other stimuli, such as audible or olfactory stimuli (23). These tests typically include behavioral ratings (reactions) to a stimulus such as a noise or a novel visual stimulus (such as a man in a hat or an umbrella opening). Because they are subjective, they also rely on the intuition and experience of the evaluator. For example, as part of the C-BARQ temperament test, the analyst rates a dog's reaction to “sudden or loud noises (e.g., thunder, vacuum cleaner, car backfire, road drills, objects being dropped, etc.)” on a 5-point Likert scale from no fear or anxiety to extreme fear (24).

In their review, Bremhorst et al. (5) discuss the current state of temperament assessment techniques, reporting that assessment tool accuracy is only 64–87% accurate according to studies (25–27). However, the ability of these tools to predict which dogs will fail is extremely variable, as low as 3% accuracy up to 85% accuracy. Overall, the tests tend to bias the results toward keeping a dog in a program; they rarely recommend releasing a dog in error. They also found that adding physiological prediction methods (such as fMRI) in combination with behavioral tests produce better accuracy (14, 28).

Within the last 5 years, researchers have been increasingly investigating prediction of a dog's suitability using these qualitative assessments. Harvey et al. show that adaptability, body sensitivity, distractibility, excitability, general anxiety, trainability, and stair anxiety can predict outcome; discusses the use of thresholds and scales to assess dogs (*n* = 1,401) (29). Additionally, Bray et al. (30) show that a decrease in body tension during an exam, a decreased reactivity to noise and prey, a decreased resistance to handling, and increased recall response in the presence of another dog are related to success.

### Toward the quantified assessment of behavior and computational ethology

In recent years, there have been calls for more universal and measurable definitions of behaviors and behavioral categories (6, 31). Based on a survey from 174 biologists and 3 biology societies, Levitis et al. define behavior as “the internally coordinated responses (actions or inactions) of whole living organisms (individuals or groups) to internal and/or external stimuli, excluding responses more easily understood as developmental changes (32).” Extending this definition, a unit measure of behavior can be defined as a specific spatio-temporal distribution of an animal's body parts (“behavior category/unit/element”) and the likelihood of those actions occurring in some order. These actions can occur sequentially or in parallel and should be related to context, aka they should exhibit “connectedness” (e.g., a tucked tail and the baring of teeth are less likely to occur in the presence of a familiar, friendly human) (33). According to Miklòsi, “the quantitative assessment of behavior [measures] the temporal distribution of these predefined behavior categories” (33). Furthermore, Miklòsi decomposes the complexity of measuring behavior as understanding behavior categories and ethograms, the temporal dynamics of behavior, splitting and lumping behaviors, arbitrary behavior measures that exist, and the importance of intra- and inter- observer agreement (33).

In the emerging field of computational ethology, the goal is to facilitate the automation of quantifying animal behaviors, particularly in ways that do not alter the animal's interactions (9, 34). Current literature focuses on using computer vision techniques for pose estimation and tracking, and the automatic behavior analysis from audio (34, 35). However, recently, Mealin et al. show extremely promising results using inertial measurement unit (IMU) data for predicting a dog's performance with 92% accuracy on the behavioral checklist (BCL), a behavior scoring system developed for and in collaboration with several US service dog organizations (23, 36, 37). Their research looks at the relationship of on-body, passive-sensing methods, specifically capturing electrocardiography and inertial data, to the activities and physiological responses exhibited during the BCL evaluation tasks (23, 36, 38). Recently, Menaker et al. (39) have started to look beyond this analysis and investigate the implications of these techniques and their ability to provide information for improving a researcher's decisions with respect to data analysis. In this paper, we approach computational ethology from the perspective of the object being interacted with, capturing actions (9) using rule-based methods (34) that are difficult to quantify using video-based techniques that rely on human coding to identify behaviors.

### Advantages of quantifying canine behavior

In *Toward a Science of Computational Ethology*, Anderson and Perona state that the “reliance on human observation to score behavior imposes a number of limitations on data acquisition and analysis” (9). They list the limitations of human observation as (1) it is slow and time-consuming, (2) it is not precise and is inherently subjective, (3) it is low-dimensional, (4) it is limited by the capabilities of human vision, (5) it is limited by what humans can describe in language, and our favorite, (6) “it is mind-numbingly boring (9).” Together, these factors can influence a study's sample size, and consequently, its statistical power and the theoretical reliability of results. Without proper considerations, the potential for various observer ascertainment biases is limited by the tools of measurement (36). In contrast, quantified methods, such as sensors and biometrics, can provide more accurate measurements that do not require tedious human observation. Data can be collected and processed with computation such as machine learning to identify patterns in the behavioral data, making temperament evaluation more efficient and effective. Sensors can also detect subtle differences in behavior that cannot be reliably observed by a human, such as the bite pressure on a toy.

### Limitations of quantifying canine behavior

There are a variety of different approaches to measuring canine activities, including body-worn sensors and video analysis (40); however, these approaches all have similar limitations. When we employ sensors to measure something, we receive valuable data about behavior, but we also introduce several constraints. First, we restrict ourselves to only what the sensors can measure (33). Aspects of the behavior that are not specifically measured by the sensors can be lost. Secondly, by adding on-body sensors or cameras in the environment, our measurements potentially introduce bias into our systems (41). For example, Clara Mancini showed how the placement of GPS on a collar changed the ranging behavior of both human and canine participants (42). Lastly, Miklòsi states that sensor systems can only recognize those categories of behavior that had been previously defined (33). In other words, sensors are unlikely to detect novel or previously unobserved behaviors.

## Experimental methodology

This section summarizes our background study described in Byrne et al. (12). Our goal was to create devices that do not require training. Fetch toys are among the most common objects used in play between humans and dogs (43). Although different breeds tend to vary in their desire to play with a ball, the retrievers that CCI raises and trains tend to enjoy toy play. Consequently, we designed new sensors in the form of common toys with which many dogs naturally engage. We built a self-contained, ball-shaped sensor approximately the size of a tennis ball consisting of food-safe silicone. We designed the instrumented ball-shaped sensors so that they could be used with or without a human so we could test for changes in interaction with the instrumented toys when humans were not directly involved in the play. Prior research indicates that companion dogs prefer play activities and interactions that involve humans over asocial interactions (44). Therefore, we wanted to be able to test whether the toys with human interaction, vs. the toys alone, could tell us anything about a dog's eventual success.

### Ethics

The methods and materials were reviewed and approved by the Institutional Animal Care and Use Committee (IACUC), the animal subject ethics board, at the authors' institution. The experimental protocol (A14109) was informed by conversations with DogStar Technologies and Canine Companions for Independence (CCI).

### Participants

We collected data from 48 dogs undergoing advanced training at the CCI facilities in parallel with Berns et al. (14), as they performed their fMRI experiments on the same cohort. All the dogs were either Labrador retrievers, golden retrievers, or lab/golden crosses. All of these dogs were purpose-bred for the CCI program. Eight of these dogs were selected for other programs outside our scope (breeding or diabetic alert) or released due to medical reasons and were removed from our cohort, leaving us with 40 dogs. Some of the dogs were still in advanced training and some were already released; however, we were blind to the outcomes of the dogs during the study to prevent bias. All dogs had basic obedience training and socialization and were between 17 and 21 months old, which is the age that the puppies transition from their puppy-raiser homes to advanced training at the CCI training centers. More detailed information on the demographics can be found in (12). At the end of our study, we learned that of the 40 dogs, 10 were released due to behavioral reasons, which varied from excessive barking to fearfulness of riding an elevator. The remaining 30 dogs successfully finished advanced training and were placed in one of five categories. They could be placed as a skilled companion, a service dog, a hearing dog, a post-traumatic stress disorder (PTSD) dog, or a facility dog, each of which has varying levels of service dog skills involved. Table 1 shows the dog outcomes for the study.

**Table 1:** Demographics and outcomes of the service dogs.

**Outcomes**	**#**
Service dog	17
Skilled companion dog	6
Facility dog	4
Hearing dog	1
PTSD dog	2
Behavioral release	10
Medical release (removed)	3
Breeders (removed)	4
Diabetic alert dog (removed)	1
**Total dogs**	**48**

A skilled companion typically has a calm temperament (as assessed by the CCI trainers), no health issues (as assessed by CCI veterinarians), and basic obedience training. They are placed with individuals who cannot manage a dog by themselves, such as children or non-independent adults with disabilities. A service dog assists with both physical tasks and provides emotional support for independent individuals with a disability. A hearing dog is trained to recognize different sounds for the hearing impaired. A PTSD dog is trained to help veterans who suffer from flashbacks or other PTSD-related conditions. A facility dog is trained to work with a professional therapist or teacher to help multiple individuals, such as at a school or therapy facility.

### Data collection process

We tested the dogs with a silicone instrumented ball (described in detail below). We traveled to CCI's National Headquarters in Santa Rosa, CA four times, testing a total of 48 dogs. We were blind to any history on the dogs other than names and ages, to reduce bias in the study. CCI trainers brought the dogs to us and took them back to the kennels after the study; we did not observe them outside of the study. We tested each dog with ten trials of each of the two ball conditions in a randomized order, with at least 30 mins of rest between:

1. Ball sensor, rolled by human (researcher)
2. Ball sensor, rolled down a ramp (machine)

We performed ten trials of each condition for each dog, for a total of twenty trials per dog. Each run of ten trials was video recorded, and after each run, the device's battery was changed, and the data and video were uploaded to determine if any loss occurred. If there was a loss, we re-ran the missing trials. Each trial was video recorded from two perspectives. One camera was near the researcher, allowing us to review the trials from the perspective of the researcher, and another camera was at the other end of the room focused on the researcher, to capture early interactions.

#### Silicone ball sensor experiment

**Figure 1:**
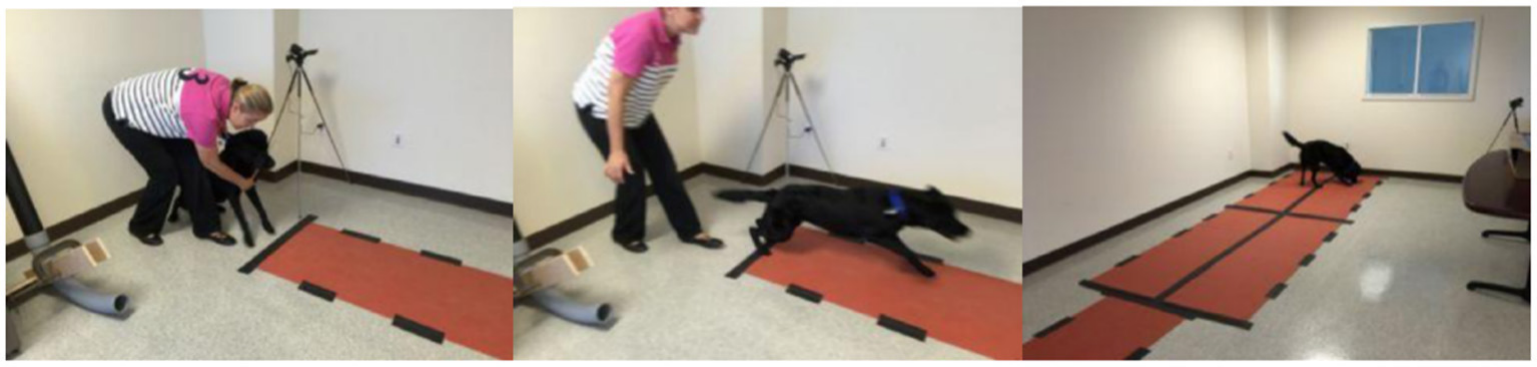
Ball-Human Sensor experiment setup. The experimenter rolls the ball, and then (left) the dog is mildly restrained for one second, and (middle) released to pursue the ball. The dog then retrieves and interacts with the ball (right).

The silicone ball sensor experiment was conducted in a closed room, with the shades drawn, to prevent distraction from trainers and other dogs. Figure 1 shows the experimental setup for the ball-human condition. Both conditions had a 15′ × 4′ wide section of the floor covered in 5 mm non-slip PVC mats so that, as the dogs ran after the ball, they wouldn't slide and hit the wall. Before and after each dog's session, we ran a magnet along the length of the ball so that we knew the exact start and finish times. After each session, the sensing board was removed from the silicone enclosure and the data was uploaded and removed.

*Condition 1—Ball rolled by a stranger*. For the first condition, a researcher unfamiliar to the dogs stood at the end of the “runway” and rolled the ball toward the other end, holding the dog back for one second before releasing the dog.

**Figure 2:**
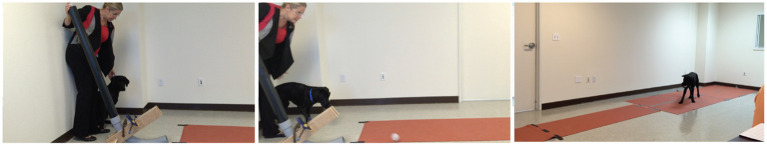
Ball-Ramp experiment setup. The experimenter drops the ball down the enclosed ramp and then (left) the dog is mildly restrained for one second, and (middle) released to pursue the ball. The dog then retrieves and interacts with the ball (right).

*Condition 2—Ball released by machine/ramp*. For the second condition, the dog started each trial next to a ramp, which was a tube with a curved end constructed of PVC pipe (shown in Figure 2). Researchers put the ball into the ramp and released it, simultaneously releasing the dog.

## Instrumented ball data collection system

**Figure 3:**
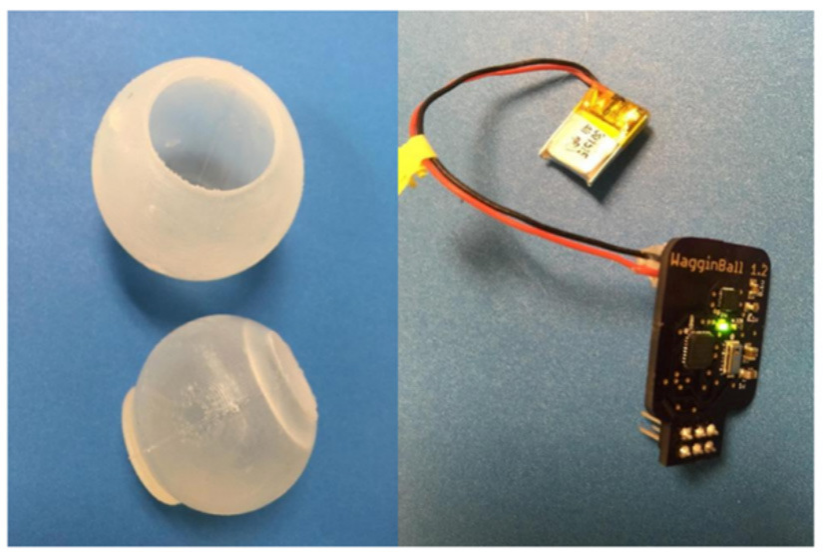
Ball sensor. left: outer ball and inner ball; right: electronics and battery that are placed inside the inner ball.

The instrumented ball sensor is composed of an inner ball and an outer ball, both molded from silicone. As shown in Figure 3, the inner ball (bottom) has an opening to accommodate inserting the electronics and has a locking mechanism to prevent the outer ball from rotating on the inner ball. The outer ball (top) has an opening to allow insertion of the inner ball. The outer ball protects the electronics and provides air space for the barometric pressure sensor to operate. When a dog bites the sensor, the air pressure inside the ball increases, and the electronics record the pressure on an SD card.

### Bite force estimations

Tools for measuring bite force continue to be developed, however, we can provide an approximation of expected bite forces per breed and by weight. Hyytiäinen et al. constructed a bite sleeve embedded with compression force sensors and found that on average German Shepherd Dogs (GSD) (*n* = 7) and Belgian Shepherd Dogs, Malinois (BSDMs) (*n* = 13) police dogs produced a median bite force of 360.4 Newton (N) and 247.0 N, respectively (45). Lindner et al. (46) use a rawhide-covered force transducer to measure bite force across 22 pet dogs that range in weight and size. On average, literature shows that dogs ranging between 11 and 23 kgs exhibited 168 N of bite force, with a range of 66–340 N. Dogs ranging between 23 and 34 kgs had a mean bite force of 180 N (range 40–367 N) and dogs heavier than 34 kgs had a mean bite force of 442 N (range 184–937 N). Ultimately, we can expect service dogs to exhibit a bite force of anywhere between 44 N and 937 N.

However, there are several important differences between measuring bite force and the work presented here. First, it is important to note that tools for capturing bite force measure at the point where the teeth meet the sensors. Our work, however, is not measuring bite force but is measuring the variability of pressure within the ball. We use this as a proxy for capturing bite strength, assuming that there is a linear relationship. Secondly, the measurements are dependent upon the type of sensors used. For example, a series of compression force sensors, such as those in the Hyytiäinen et al. paper, will provide high granularity force measurements across a sleeve, providing a range of localized measurements where presumably the force closer to the temporomandibular joint (fulcrum) is higher than the forces exhibited by the canine teeth; whereas a single-dimension transducer, such as the Lindner paper, will provide an average bite force across the rawhide “plate.” We did not perform a full calibration of our instrumented ball to capture the mapping of pressure to force, however, the lack of calibration doesn't affect the model's ability to discriminate dogs' performance. This calibration will be included in future work.

### Instrumented ball implementation

The electronics consist of a custom printed circuit board that we designed. A barometer and a 9-axis inertial measurement unit (IMU) were integrated into the board. The barometer is a sensor that measures the changes and variations in internal pressure based on the ambient internal air pressure. We chose this barometer specifically for its calibration specifications—the value provided for barometric and atmospheric pressure accounted for pressure sensor linearity and the variability in ambient temperature, such as a city's altitude^1^. Data was collected in kiloPascals (kPa).

Additionally, our electronics incorporated an IMU device to capture the force and angular movement exhibited on the ball. Specifically, the IMU collected changes in X, Y, and Z values of the accelerometer, gyroscope, and magnetometer. The accelerometer in the IMU measured the rate of change in velocity, which allowed us to capture some “kill behaviors” (shaking toy) as well as characterize the intensity and duration of the dog's play behaviors. The IMU's gyroscope measured orientation and angular velocity, and let us quantify movement “gestures,” as well as detect a rolling ball. The magnetometer in the IMU measured the strength and direction of magnetic fields and gave us the opportunity to perform a sync trigger with a small magnet useful for synchronizing our data collection with the video recording.

Lastly, the translucence of the ball allows researchers to observe the green light inside, indicating that the board has power. We built two new boards for the ball sensors and six new silicone balls to prepare for our first test at CCI.

## Analysis methodology

Our previous analysis focused on predicting whether a service dog would be placed in advanced training; however, for this study, we were interested in constructing hypotheses about the differences and relationships of toy interactions between successfully being placed as a service dog or not. Using the data from our final cohort of 40 dogs, we start by engineering features and generating a set of summary statistics of the trials. Next, we used a general linear model (GLM) with a binomial probability distribution over a dog's individual trial summary features to explore which interactions were more likely to be exhibited by service dogs who are placed in homes. Additionally, to gain more insight into how these relationships change with respect to each feature, we estimate service dog success given specific instrumented ball interactions. This analysis was conducted in RStudio (47).

### Feature engineering of the instrumented ball

One primary method for automatic behavior classification is to use rule-based methods for feature engineering. The disadvantages of this method according to Egnor and Branson (34) are that rule-based detection is difficult to tune; it may depend only on a minimal number of features; it fails for more complicated behaviors; and it rarely generalizes well. Here, we are more interested in looking at the base features, constructing summaries of these interactions as opposed to building out trajectories over time. The primary advantage of feature engineering is that it allowed us to simplify our model, making it faster to run and easier to understand and maintain over time. Feature engineering allowed us to also understand the underlying behaviors of the different dog classes and therefore provide further insight into how these tools can benefit working and service dog programs.

**Figure 4:**
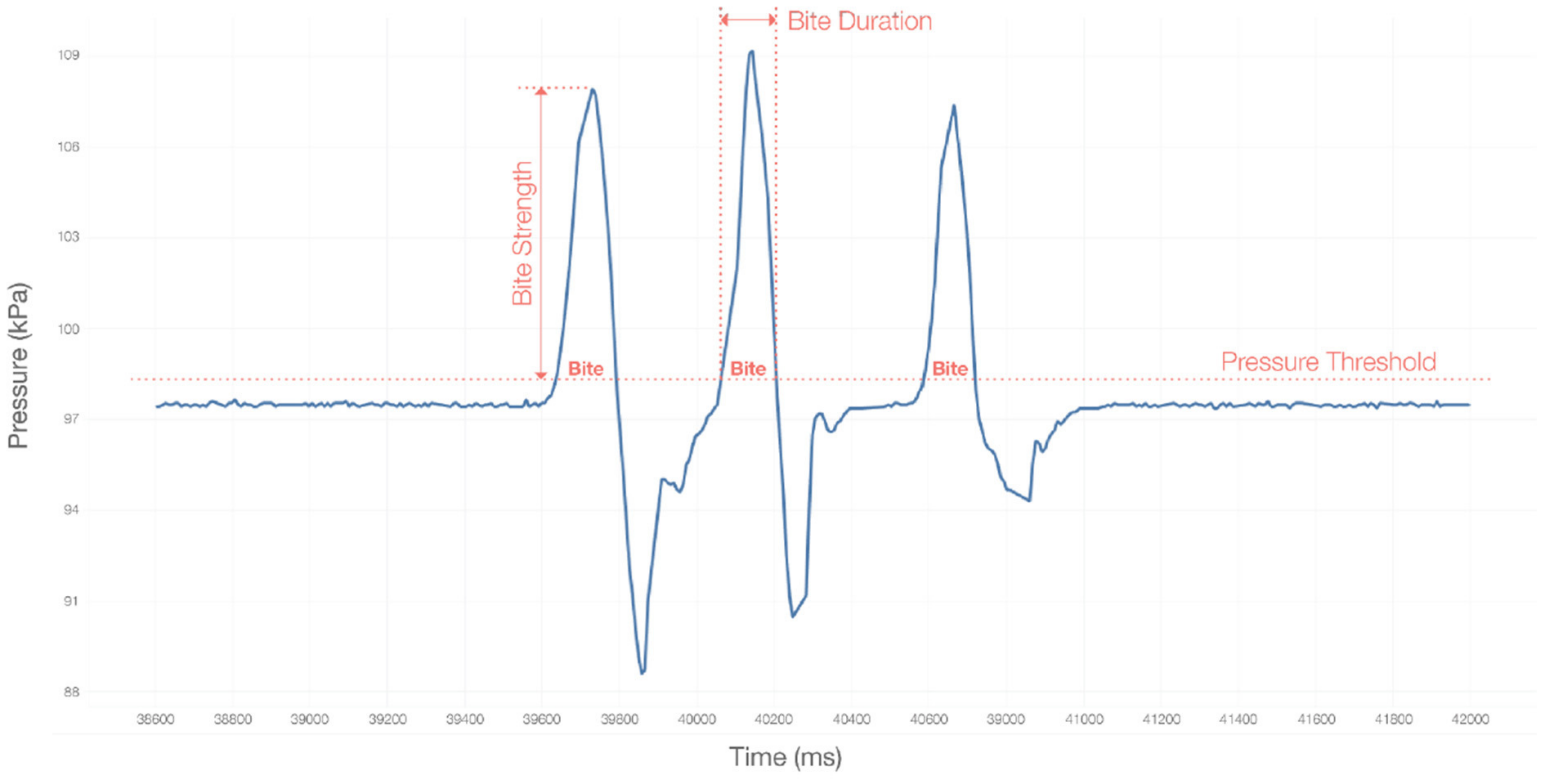
A visual description of the engineered bite.

We constructed 22 features from the raw data of each of the ball conditions. We constructed these features from observations during previous generic canine interactions. To generate the trial summary statistics, we calculate a core set of features and then determine the average, maximum, and total measurements of these features during each trial. These features are visualized in Figure 4.

For the instrumented ball, our features were:

- Interaction Time: the amount of time required for a single trial, from the time the ball rolled out of the ramp or hand until the dog retrieved the ball to the handler.
- Number of Bites: the number of times the pressure crosses a threshold during each trial in a dog's session.
- Average bite strength: the peak pressure throughout the duration of a bite.
- Average bite duration: the amount of time between the beginning and end of a bite.
- Average bite frequency: number of bites in a trial, divided by the length in seconds of that trial.
- Average bite RMS: the root mean square of all pressure samples throughout the duration of a bite.

### General linear model

The trial summary statistics data is an unbalanced dataset, where the number of trials was between 8 and 10. The general equation for GLM is:


$$\begin{array}{l} y_{i}=\beta_{0}+\beta_{1}X_{1i}+\beta_{2}X_{2i}+...+\beta_{k}X_{ki}+\;\varepsilon_{i}, \end{array}$$

where the binary response variable, *y*_*i*_, *i* = 1, is modeled by a linear function of a set of k explanatory variables, *X* = (*X*_1_, *X*_2_, ... *X*_*k*_), plus an error term. β represents the coefficients, or weights, for their associated variable *X*.

**Figure 5:**
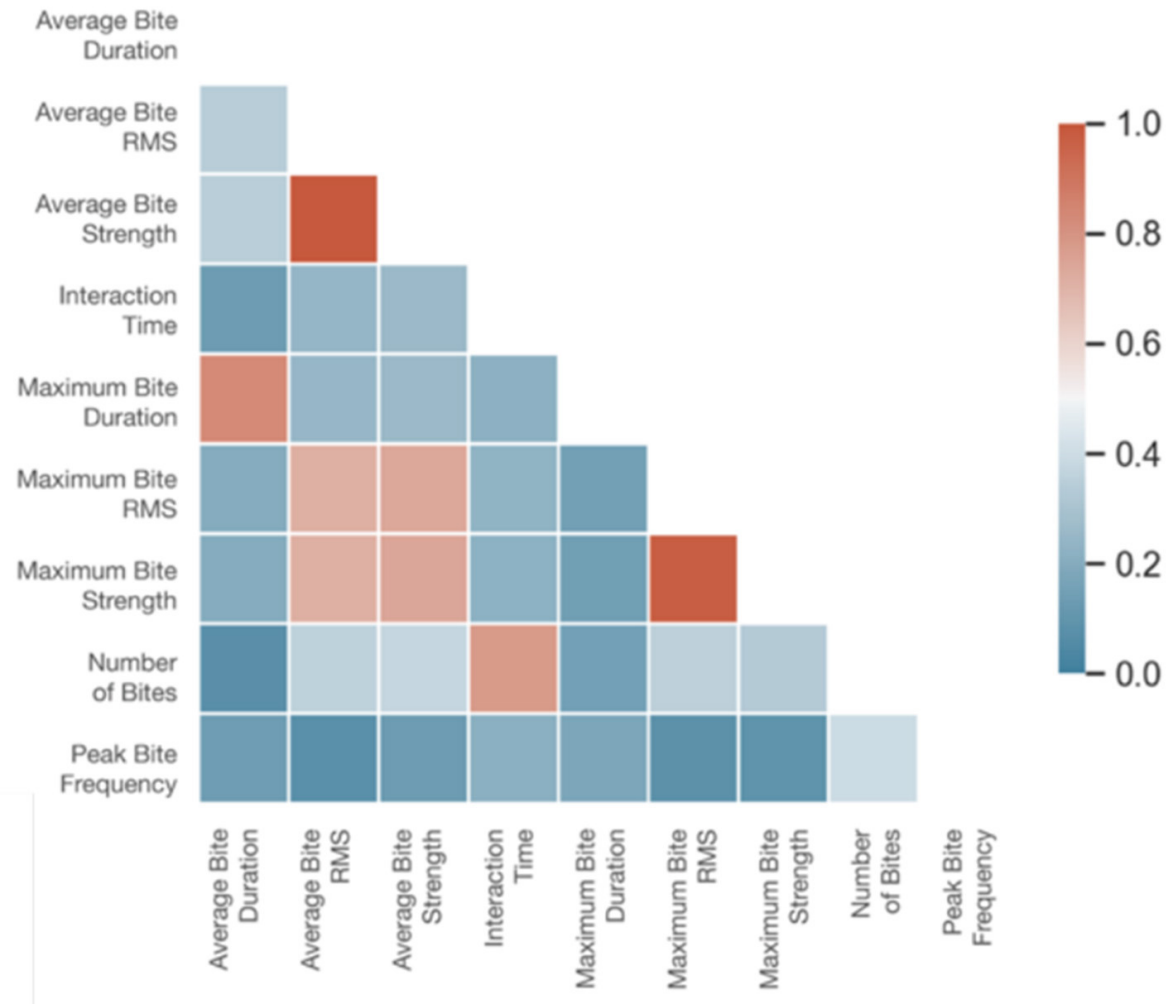
Correlation matrix of CCI features collected by the instrumented ball (modeling feature importance with respect to class).

Before conducting our analysis, we used a heatmap correlation matrix (shown in Figure 5) to identify the highly correlated features and determine which features lack multicollinearity. We then remove the variables with high inter-correlations and perform the analysis with the five following independent, explainable variables:

1. Average bite duration: Average bite duration within a trial.
2. Average bite strength: Average peak pressure throughout the duration of a bite.
3. Interaction time: the amount of time required for a single trial, from the time the ball rolled out of the ramp or hand until the dog retrieved the ball to the handler.
4. Peak bite frequency: The maximum number of bites in a trial, divided by the length in seconds of that trial.
5. Condition: This feature can either be “human” or “ramp”.

We then calculate the odd ratios for each feature, which provide us with an estimate and the confidence intervals of a relationship between our binary outcomes (48).

### Estimated marginal means

We use the output of the GLM to calculate the estimated marginal means, and their confidence intervals. The estimated marginal means provides us with the mean response for each class, adjusted for each of the covariates (49), and visualizes the deltas between classes.

To calculate the estimated conditional expectation of Y we use:


$$\begin{array}{l} E\left[Y\;=\;y\;|X_{i}=\;x,\;X_{condition}\right], \end{array}$$

where *Y* is the outcome and *X* is the expectations of the outcomes given the independent variable on the x-axis and the condition. We provide a corresponding plot to show the expectation of predicted outcome over the range of each feature.

## Results

**Figure 6:**
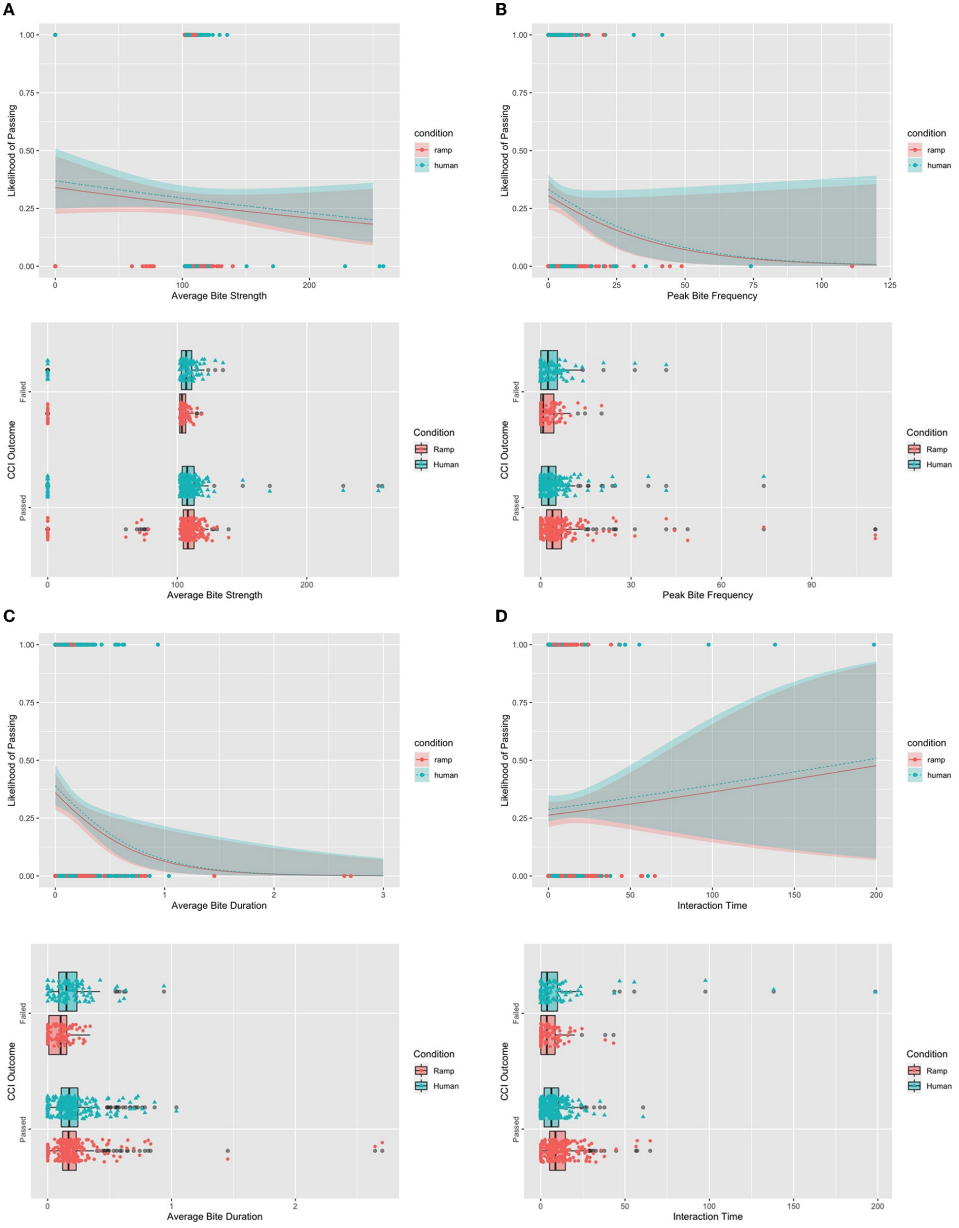
Likelihood and distribution plots for each feature. **(A)** Top: The likelihood of passing the CCI criteria using average bite strength plotted by condition. Bottom: Distribution of average bite strength for each condition plotted against CCI outcome. **(B)** Top: The likelihood of passing the CCI criteria using peak bite frequency plotted by condition. Bottom: Distribution of peak bite frequency for each condition plotted against CCI outcome. **(C)** Top: The likelihood of passing the CCI criteria using average bite duration plotted by condition. Bottom: Distribution of average bite duration for each condition plotted against CCI outcome. **(D)** Top: The likelihood of passing the CCI criteria using interaction time plotted by condition. Bottom: Distribution of interaction time for each condition plotted against CCI outcome.

In total, we ran 960 trials (10 trials ^*^ 2 conditions ^*^ 48 dogs). We discard the 160 trials collected from the dogs removed from our cohort. Out of the remaining trials, the dogs didn't interact with the instrumented ball for 41 of the trials in the “Machine/Ramp” condition and 36 of the trials in the “Human” condition. We report those as zeros in the data. For example, in Figure 6, you will see an average bite strength of zero in the plots and this refers to zero interaction.

### Adjusted odds ratios

Adjusted odds ratios provide an interpretable measure to the general linear model output. These adjusted odds ratios provide insight into the strength of correlation of a feature to an outcome while also controlling for other predictor variables. Thus, we use this adjusted odds ratio to discuss feature importance. The “Intercept” gives us the “base” log odds, which is the log odds when all the variables are 0, and the coefficients that are associated with a variable give us how much that log odds increase every time the corresponding variable goes up by 1 unit. Per the results shown in Table 2, both Average Bite Duration *[Adjusted OR* = *0.12, Pr(*>*|z|)* = *0.00666]* and Peak Bite Frequency *[Adjusted OR* = *0.97, Pr(*>*|z|)* = *0.07264]* show decreased odds with the likelihood of becoming a service dog. Across all four features we see that the human condition is more likely to provide us with higher and potentially more precise estimates of passing the criteria for being a service dog at CCI.

**Table 2:** Adjusted odds ratios of the general linear model for CCI outcomes.

	**Adjusted OR**	**2.5%**	**97.5%**
(Intercept)	0.88	0.56	1.40
Average bite strength	1.00	0.99	1.00
Peak bite frequency	0.97	0.93	1.00
Average bite duration	0.12	0.02	0.49
Interaction time	1.00	0.99	1.00
Condition (Human)	1.13	0.80	1.60

### Distributions and their estimated marginal means

To understand the influence of these features further, we display the estimated conditional expectation, or likelihood, of being placed as a service dog for each feature: Average Bite Strength (in Figure 6A, Top), Peak bite frequency (in Figure 6B, Top), average bite duration (in Figure 6C, Top), and interaction time strength (in Figure 6D, Top). The line in these plots shows the estimation of a dog passing against a feature, while the ribbon displays the 95% confidence intervals associated with those estimations, and the points display the data points of each class. Per the legend, blue features refer to the Ball-Human condition, while the red features refer to the Ball-Ramp condition. Below each likelihood plot, we also provide a boxplot displaying the distributions of each feature across conditions and CCI outcomes (see in Figures 6A–D, Bottom).

We also provide the mean, minimum, and maximum values of the independent features for each condition. Average values across the entire cohort of dogs are reported, as well as the mean, min, and max values for the dogs who succeeded in being placed (pass) and the dogs who did not succeed (fail). Values for the Ball-Ramp condition can be found in Table 3 and values for the Ball-Human condition can be found in Table 4. The primary goal of these tables is to give insight into what the range of values looked like when the dogs *were* interacting with the ball. Therefore, we did not include trials where the dog did not interact with the ball in this analysis. These “zero-interaction” trials, however, were included in the estimated marginal means analysis.

**Table 3:** Mean, min, and max values for each independent feature for the ball-ramp condition (feature average and by outcome; does not include trials with zero interactions).

	**Avg bite strength (kPa)**			**Peak bite freq [max #/trial time (s)]**			**Avg bite duration (s)**			**Interaction time (s)**		
	**Avg**	**Pass**	**Fail**	**Avg**	**Pass**	**Fail**	**Avg**	**Pass**	**Fail**	**Avg**	**Pass**	**z**
Mean	108.07	108.96	105.97	6.82	7.16	3.44	0.21	0.24	0.14	10.95	12.11	8.19
Min	60.33	60.33	101.83	0.00	0.00	0.00	0.01	0.01	0.01	0.01	0.01	0.02
Max	139.61	139.61	118.65	111.11	111.11	20.20	2.70	2.70	0.34	64.95	64.95	43.18

**Table 4:** Mean, min, and max values for each independent feature for the ball-human condition (feature average and by outcome; does not include trials with zero interactions).

	**Avg bite strength (kPa)**			**Peak bite freq [max #/trial time (s)]**			**Avg bite duration (s)**			**Interaction time (s)**		
	**Avg**	**Pass**	**Fail**	**Avg**	**Pass**	**Fail**	**Avg**	**Pass**	**Fail**	**Avg**	**Pass**	**Fail**
Mean	111.04	111.87	109.13	5.56	4.58	4.44	0.23	0.23	0.21	9.77	8.64	12.37
Min	101.86	101.93	101.86	0.00	0.00	0.00	0.01	0.01	0.01	0.01	0.01	0.01
Max	258.19	258.19	135.27	74.07	74.07	41.67	1.04	1.04	0.94	198.50	60.72	198.50

#### Average bite strength

Average bite strengths for interactions with the Ball-Ramp condition range between 60 and 139 kPa, reporting a mean strength of 108 kPa. For trials interacting with the Ball-Human condition, on average the dogs exhibited a minimum of 102 kPa bite strength and a maximum of 258, where the mean bite strength hovered around 111 kPa.

Looking across the outcomes of each dog, we see that dogs who do not get placed, who fail, demonstrate a range of average bite strength, within the Ball-Ramp condition, from 102 to 119 kPa, with a mean value of 106 kPa when they interact with the toy. For the Ball-Human condition, however, their average bite strengths range from 101 to 135 kPa, with a mean value of 109 kPa, and demonstrate a wider range of values than in the Ball-Ramp condition when they interact.

Dogs who are placed in a role have a wider range of average bite strength when they interact in the Ball-Ramp condition, from 60 to 140 kPa, with a mean bite strength of 109 kPa. In the Ball-Human condition, we find that the minimum values of bite strength stay around 102 kPa, similar to the dogs who fail, however, their maximum average bite strength values within a trial can go up to 258 kPa when the dog interacts with the instrumented ball. Their mean bite strengths are also slightly higher than the dogs who fail, at 112 kPa.

The distributions at the bottom of Figure 6A visualize some of the trends. The plot shows higher ranges of bite strength when the dogs are interacting with a human and that the bite strength shows higher variability for dogs who are placed. The top of Figure 6A shows a downward slope in the likelihood of passing for dogs who have a higher bite strength, suggesting that dogs who have higher bite strength will not become active service dogs.

#### Peak bite frequency

When the dogs interact with the toys during the trial, the average peak bite frequencies in the Ball-Ramp condition range between 0 and 111 max bites/trial time (s), with a mean frequency of 7 max bites/trial (s). Contrastingly, the dogs exhibit average peak bite frequencies for the Ball-Human condition between 0 and 74 max bites/trial (s), however their mean values are similar to the Ball-Ramp condition, around 6 max bites/trial (s). The range in general for peak bite frequency in the Ball-Human condition appears to have less variability. Interestingly, the zeros as minimum values here demonstrate that there is a quick but minimal interaction with the toy, as showcased by the low values in average bite duration and interaction time.

For dogs who do not get placed, their peak bite frequencies in both the Ball-Ramp and Ball-Human conditions show ranges of 0–20.20 and 0–41.67 max bites/trial (s), respectively. The Ball-Human condition shows more variability in dog interaction, as their mean values are slightly higher at 4 max bites/trial (s).

The dogs who do get placed show peak bite frequencies of 0 to 111 max bites/trial (s) in the Ball-Ramp condition, with a mean peak bite frequency of 5 max bites/trial (s). For the Ball-Human condition, we find lower values, ranging from 0 to 74 max bites/trial (s), with a mean value of 5 max bites/trial (s). While the means of the two conditions are the same, the variability differs.

Overall, the variability between dogs who are placed and dogs who do not get placed is very different. Interestingly, in the distribution plot of Figure 6B we see that when the dog does not get placed, the distribution is wider in the Ball-Human condition, while the distribution is wider in the Ball-Ramp condition when the dog is placed. In Figure 6B's estimated marginal means plot, we see that the likelihood of passing the CCI criteria decreases as peak bite frequency increases.

#### Average bite duration

On average, the average bite duration when a dog interacts with the instrumented toy in the Ball-Ramp condition, we see a range between 0.01 and 2.7 s, with a mean time of 0.21 s. The average bite duration ranges in the Ball-Human condition go from 0.01 to 1.04 s with a mean score of 0.23s—slightly higher than the Ball-Ramp condition.

Looking at the dogs who do not get placed, we see that when the dogs are interacting with the toys in the Ball-Ramp condition, their bite durations last on average between 0.01 and 0.34 s with a mean time of 0.14 s. In the Ball-Human condition, the bite durations that the dogs exhibit on average range from 0 to 1.04 s, with a mean value of 0.23 s.

The average bite durations for dogs who do get placed demonstrate do not appear much different. The dogs who interact with the ball in the Ball-Ramp condition showcase ranges from 0 to 2.7s with a mean bite duration of 0.24 s; meanwhile, the average bite durations in the Ball-Human condition are lower, ranging from 0.01 to 1.04 s with a mean value of 0.23 s.

In the distribution plot at the bottom of Figure 6C we see that the distributions across conditions for dogs who pass are similar. However, we see differences in average bite duration within the set of dogs who do not get placed and between the dogs who get placed and the dogs who do not. Additionally, as we can see in Figure 6C, as the average bite duration increases over time, the estimated likelihood of that dog passing reduces.

#### Interaction time

Lastly, we look at the differences in interaction time. On average, we find that active trials in the Ball-Ramp condition had mean interaction times of 10.95 s, with a range of 0.01–64.95 s. The Ball-Human condition shows a slightly lower mean interaction time, 9.77 s, but wider spread from 0 to 198.5 s.

Diving into the dogs who did not get placed, we see that the average interaction time during the Ball-Ramp condition extends from 0.02 to 43.18 s with a mean of 9.18 s. Contrastingly, we see that the interaction times range from 0.01 to 198.5 s in the Ball-Human condition and have a mean value of 12.37 s.

The differences in interaction times for dogs who were placed lie in the mean values of the distribution. In the Ball-Ramp condition, the interaction times ranged from 0.01 to 64.95 s with a mean interaction time of about 12 s. The interaction times for the Ball-Human condition range from 0.01 to 60.72 s and have a mean value of 8.64 s.

As shown in Figure 6D, the mean values and ranges within conditions are inverted between dogs who are placed and dogs who are not. Furthermore, in the estimated marginal means plot of Figure 6D, we see an upward trend in interaction time for dogs who are more likely to become a service dog. However, the high variance exhibited by the confidence intervals suggests a lack of confidence in that estimation.

## Discussion

The primary goal of this research was to assess whether instrumented toys, and computation in general, could provide more insight into what a successful service dog means. The instrumented ball showed promise as a method for helping characterize temperament by using rule-based methods for engineering features that are difficult to quantify using other techniques. Previously, we showed that these toys can predict whether a service dog is placed, but not why our predictions are successful.

In this study, we analyze the engineered features within each trial using a GLM with a binomial probability distribution to determine which features and interactions were more likely to be exhibited by successfully placed service dogs. Furthermore, we estimate the likelihood of service dog success and plot how those change as the interactions change. We identify that average bite duration and peak bite frequency contribute the most to our understanding of service dog suitability using these toys, as their odds ratios show some level of significance. One hypothesis as to why average bite duration and peak bite frequency is important is that service dogs are taught to bite precisely, such as biting to pull a sock off a foot, and we could potentially be capturing the differences in dogs who treat the ball as a play object as opposed to a “thing to precisely interact with.” These findings, however, support previous studies which have shown chewing as an important feature for predicting service dog placement (30).

Additionally, it is important to highlight that within our characterization of a dog's average bite duration and peak bite frequency, the distribution of the data has more variability in the “ramp” condition for dogs who “passed” or were deemed suitable for service dog work. Furthermore, the distribution of data across all four features shows the mean scores are slightly lower when interacting with a human. This finding suggests that dogs who show less interest in playing with a human are more likely to successfully be placed.

Except for interaction time, the dogs who do not get placed as service dogs exhibit a narrower range of distributions for each independent feature. Reflecting on the study, interaction time is interesting because two of the 13 dogs who failed played “keep away” or became aggressive when the humans rolled the ball, extending the interaction time. This behavior again suggests that the relationship with any human is nuanced.

In the past, hesitation has been expressed at the inclusion of both conditions as it can be tedious to run multiple studies; however, the distribution plots show the importance of having a human involved in the study implementation as well as not having a human participating. Again, this appears to be supported by literature.

### The relationship of correlation and prediction

Tying this analysis back to our original work, we discuss the individual feature performance of the instrumented ball interactions (12). In the prior prediction analysis, we summarize the individual trial data used in this analysis to generate statistics across a dog's entire session. Using a subset of these features, we were able to predict a dog's suitability with 87.5% accuracy.

While investigating the validity of our prior research, we showed that variations of the number of bites and average bite duration independently provided between 72.5 and 82.5% accuracy of predicting whether a dog would be suitable for placement within a home, as shown in Table 5.

**Table 5:** Individual feature performance metrics in predicting service dog placement (adapted for relevance).

	**% Accuracy**	**Precision**	**Recall**	***F***	**MCC**	**AUC**
Max # bites—ball ramp	82.5%	0.821	0.825	0.803	0.480	0.798
Avg # bites—ball ramp	80%	0.792	0.800	0.766	0.385	0.780
Max # bites—ball human	75%	0.563	0.75	0.643	0.000	0.500
Avg bite duration—ball ramp	72.5%	0.679	0.725	0.690	0.131	0.510

The analysis presented in this paper supports the prior prediction analysis. As a result, we can provide insight into why our computational play-based interactions are indicative of service dog suitability. In particular, we can say that as a dog takes longer to bite on their instrumented balls, the likelihood of that dog being placed in a home reduces significantly.

### Limitations

We used f2, a power analysis method for general linear models, within RStudio's pwr package to calculate how many dogs we would need to run to achieve statistical significance. With the 5 coefficients we use in the GLM, 0.5 statistical significance, a small expected effect size, and a goal to achieve a 0.80 power level, we would need to collect ~641 data points. Since we use both ramp and human conditions, we collect 20 data points per dog, which equates to 33 (to be inclusive) dogs worth of data for each research question. Our research achieved these significance numbers for the Service Dog analysis.

The majority of our limitations lie with our hardware and our software. Our initial experiments highlighted situations in which the sensors could fail and allowed us to make them more robust. Initial problems included:

#### Silicone failure

Initially, dogs could bite hard enough to puncture the silicone and consequently damage the electronics inside. We experimented with different densities and thicknesses of silicone and discovered that a harder silicone prevented the ball from being punctured. We used the same silicone density and hardness for all of the testing in the CCI study.

#### Hardware failure

In general, the hardware did not fail unless the ball was punctured. However, we did have a few instances of the battery being unplugged, or the SD card being ejected by bites that perfectly aligned with those junctures. We solved this by wrapping the inner electronics in soft fabric to cushion them and to keep them from moving around inside the inner ball.

#### Inner ball rotation

Some of the harder-biting dogs were able to compress the outer ball enough that the inner ball could rotate inside of it, allowing the opening to be exposed. This problem was exacerbated by the fact that the dog's saliva could lubricate the two pieces of the ball to allow them to slip more easily, so dogs that interacted longer were more likely to rotate the inner ball. We describe a fully-contained ball in our future work.

### Future directions

#### Self-enclosed

We completed all testing with the original nested-ball sensor design for continuity. However, we have been designing and experimenting with a fully-enclosed silicone ball that would be superior for “real world” use. This improvement would minimize damage to the internal components and speed testing, because the ball would not need to be disassembled to upload the data and change the battery. It would also allow for us to capture data wirelessly, transmitting all of the data reliably.

#### Additional testing to boost accuracy and increase generalizability

To further refine and verify Smart Toys, we intend to continue testing what we have discovered on new cohorts of dogs. As mentioned earlier, the cohort we tested required training to enter an fMRI and to participate in being scanned. Given that the normal graduation rate is 40% and that the graduation rate of this set was 75%, we wonder if the ball could help reject dogs in the average cohort of CCI candidates even more accurately than suggested in the testing above. Investigating dogs in other service programs would allow us to extend the generalizability of this framework to a wider class of working dogs.

#### Leveraging activity recognition to understand the quality of each interaction and how it changes over time

Our goal for this study was to initially examine what it would take to predict the success and failure of service dogs. Opportunities exist to dive deeper and investigate how a canine's interactions vary across different temperaments. For example, do dogs with varying levels of reactivity or attachment have different bite patterns? We are also intrigued by the possibility of using changes in our features to determine the ongoing health of an individual dog or their ability to perform their duty on a given day.

#### Exploring the relationship of familiar humans and strangers

We know that there are differences in which portions of the dog's brain activate when seeing humans of varying familiarity. The results above show evidence that play interactions are altered when a human is involved and their importance to successful placement as a service dog. Given the socialization strategies used by most service dog organizations and the fact that these dogs are placed in new homes, it would be interesting to study how varying levels of familiarity influence quantified play behavior.

## Conclusion

In this study, we have shown that play-based interactions measured using an instrumented ball can quantify a canine's object-play behavior. We also constructed a novel methodology for building and evaluating predictive models that forecast the suitability of puppies successfully completing advanced training. Exploring outputs from the sensors allowed us to identify various features that are more valuable for the prediction of a service dog and we used these features to validate our original predictive model demonstrating 87.5% accuracy. Furthermore, we discuss why these models are effective models that could be significant for helping service dog organizations reduce the cost of training dogs, increase the efficiency of their programs, and enable trainers to spend more time developing dogs with temperaments more suitable for service dog careers.

## Data availability statement

The datasets presented in this article are not readily available because we do not have approval to share this dataset with others. Requests to access the datasets should be directed to cearabyrne@mit.edu.

## Ethics statement

The animal study was reviewed and approved by IACUC, Georgia Institute of Technology. Written informed consent was obtained from the owners for the participation of their animals in this study.

## Author contributions

MJ, TS, and CB developed the experimental protocol and designed the toys. MJ and CB ran experiments. CB conducted the data analysis under the advisement of MJ and TS. All authors contributed to the article and approved the submitted version.

## Funding

This material is based upon work supported by the Army Contracting Command and DARPA under Contract No. W911NF-14-C-0094 to Dog Star Technologies, LLC.

## Conflict of interest

The authors declare that the research was conducted in the absence of any commercial or financial relationships that could be construed as a potential conflict of interest.

## Publisher's note

All claims expressed in this article are solely those of the authors and do not necessarily represent those of their affiliated organizations, or those of the publisher, the editors and the reviewers. Any product that may be evaluated in this article, or claim that may be made by its manufacturer, is not guaranteed or endorsed by the publisher.

## Author disclaimer

Any opinions, findings and conclusions or recommendations expressed in this material are those of the authors and do not necessarily reflect the views of the Army Contracting Command and DARPA.

## Footnotes

^1^Miniature I2C Digital Barometer.16.
